# Altered Levels of Acetylcholinesterase in Alzheimer Plasma

**DOI:** 10.1371/journal.pone.0008701

**Published:** 2010-01-14

**Authors:** María-Salud García-Ayllón, Iolanda Riba-Llena, Carol Serra-Basante, Jordi Alom, Rathnam Boopathy, Javier Sáez-Valero

**Affiliations:** 1 Instituto de Neurociencias de Alicante, Universidad Miguel Hernández-CSIC, San Juan de Alicante, Spain; 2 Centro de Investigación Biomédica en Red sobre Enfermedades Neurodegenerativas (CIBERNED), Madrid, Spain; 3 Servicio de Neurología, Hospital General Universitario de Elche, Alicante, Spain; 4 Department of Biotechnology, Bharathiar University, Tamil Nadu, India; University of Oulu, Finland

## Abstract

**Background:**

Many studies have been conducted in an extensive effort to identify alterations in blood cholinesterase levels as a consequence of disease, including the analysis of acetylcholinesterase (AChE) in plasma. Conventional assays using selective cholinesterase inhibitors have not been particularly successful as excess amounts of butyrylcholinesterase (BuChE) pose a major problem.

**Principal Findings:**

Here we have estimated the levels of AChE activity in human plasma by first immunoprecipitating BuChE and measuring AChE activity in the immunodepleted plasma. Human plasma AChE activity levels were ∼20 nmol/min/mL, about 160 times lower than BuChE. The majority of AChE species are the light G_1_+G_2_ forms and not G_4_ tetramers. The levels and pattern of the molecular forms are similar to that observed in individuals with silent BuChE. We have also compared plasma AChE with the enzyme pattern obtained from human liver, red blood cells, cerebrospinal fluid (CSF) and brain, by sedimentation analysis, Western blotting and lectin-binding analysis. Finally, a selective increase of AChE activity was detected in plasma from Alzheimer's disease (AD) patients compared to age and gender-matched controls. This increase correlates with an increase in the G_1_+G_2_ forms, the subset of AChE species which are increased in Alzheimer's brain. Western blot analysis demonstrated that a 78 kDa immunoreactive AChE protein band was also increased in Alzheimer's plasma, attributed in part to AChE-T subunits common in brain and CSF.

**Conclusion:**

Plasma AChE might have potential as an indicator of disease progress and prognosis in AD and warrants further investigation.

## Introduction

Alzheimer's disease (AD) is the leading cause of dementia in the aged population. The main neuropathological changes associated with AD are β-amyloid plaque accumulation, neurofibrillary tangle formation and substantial synaptic and neuronal loss in critical brain areas. In particular, there is good evidence of cholinergic dysfunction in the AD brain. Acetylcholinesterase (EC 3.1.1.7; AChE), the enzyme chiefly responsible for the inactivation of cholinergic neurotransmission, is consistently decreased in the AD brain [1], [2]. Despite this overall decrease, levels of AChE are increased around β-amyloid plaques [3], [4] and it has been proposed that AChE may play a role in β-amyloid fibrillogenesis [5], [6]. In this context, the altered expression pattern of AChE species in the AD brain is also of particular interest. AChE exhibits a complex structural polymorphism depending upon its different cellular distribution, whose significance is highly intriguing [7]. The different molecular forms of AChE are altered in AD, with a decrease in the major AChE tetramers (G_4_) - probably the cholinergic species - and subtle increase in minor light forms (dimers, G_2_, and monomers, G_1_) [8], [9]. Interestingly, the activity of the light forms appears to increase in the most severely affected cases [10].

While the level of AChE and its molecular species are altered in the AD brain, AChE activity in the cerebrospinal fluid (CSF) has also been measured in assessing the pathophysiology of AD. The emerging consensus is that total CSF-AChE levels decrease modestly as dementia progresses. The proportion of G_1_ is enriched, but the changes are not specific for early diagnostic and prognostic utility [11]–[14]. Nonetheless, measurement of AChE levels in AD might have some value in monitoring disease progression and is still of interest due to increasing evidence linking β-amyloid processing and AChE activity.

Although the CSF is a more relevant source to examine diagnostic markers for AD, plasma offers a distinct advantage as it is more easily accessible for clinical use in monitoring disease progression and therapeutic interventions. Thus, many studies have assessed plasma AChE as a marker for AD, with limited success and reliability [15]–[19]. A major disadvantage encountered is that human plasma is rich in a second enzyme capable of hydrolysing acetylcholine - butyrylcholinesterase (EC 3.1.1.8; BuChE), while only a minor amount of AChE is present [20]. As a result many previous and current reports on AChE activity in plasma in several neurological and neuropsychiatric disorders have over-estimated plasma AChE levels due to the cross catalytic activity of BuChE. Attempts to evaluate plasma AChE by immunoassay [21]–[24] have used antibodies raised against only the major AChE forms from brain or erythrocytes and do not react with all AChE species [25]–[28]. In particular, the antibodies display low affinity for monomers [14], [29], [30]. As a result, levels of plasma AChE are often under-estimated when measured by immunoassay. Although extensive studies have been conducted in the last three decades, a suitable assay to measure plasma AChE levels is still lacking.

In this study, we have measured AChE activity in human plasma, eliminating BuChE interference by prior BuChE-immunoprecipitation. We have compared AChE activity levels and molecular forms in normal control subjects to the levels from human BuChE silent individuals. We have compared the different molecular forms and subunit banding pattern of AChE by SDS-PAGE under reducing conditions followed by Western blotting using different anti-AChE antibodies, and glycoform patterns by lectin-binding analysis. Plasma AChE has been compared to AChE from human liver, red blood cells (RBCs), CSF and brain. Finally, we have investigated whether AChE levels are altered in the plasma of AD patients compared to normal age-matched individuals.

## Methods

### Human Samples and Tissue Preparation

This study was approved by local ethics committees and was carried out in accordance with the Declaration of Helsinki. Plasma samples were collected in heparinized tubes at the Hospital General Universitario de Elche (Spain) and separated from whole blood by centrifugation at 3000×g for 15 min at 4°C, aliquoted and frozen at −80°C until use.

Patients with probable AD [11 females/3 males, 77±2 yrs (mean± SE)] had to fulfill the criteria for the clinical diagnosis of probable AD established by the Working Group of the National Institute of Neurological and Communicative Disorders and Stroke (NINCDS) and the Alzheimer's Disease and Related Disorders Association (ADRDA) [31]. None of the cases selected had previously received cholinesterase inhibitor treatment. The disease duration was 27±4 months, and the severity of dementia, evaluated using Mini-Mental State examination [32], was 19±1. For the non demented control (ND) group, age and gender-matched healthy volunteers [12 females/3 males, 76±1 yrs (mean ± SE)] without history, symptoms or signs of psychiatric or neurological disease were enrolled.

Serum samples were also collected from three healthy BuChE silent subjects (males) from the Vysya community in India (30±5 years). The frequency of homozygous silent BuChE (a substitution of leucine 307 by proline) in the Vysya community is 1 in 24, a value 4000-fold higher than the frequency of homozygous silent BuChE in European and American populations [33]. These individuals are completely deficient in BuChE but have only minor abnormalities in clinical test results [34], [35].

For the initial characterization of AChE from plasma and red blood cells, samples from healthy subjects [3 females/3 males, 46±4 yrs] were employed. Red blood cells (RBCs), separated from plasma by centrifugation, were washed three times with 154 mM NaCl, 5.4 mM EDTA, 5 mM phosphate buffer, pH 7.5, lysed by suspension in 20 volumes of 5 mM phosphate buffer, pH 7.5, and membranes collected for AChE extraction by centrifugation at 10,000×g for 30 min at 4°C. CSF was collected from non-demented controls at the Hospital Universitario San Carlos in Madrid (obtained by lumbar puncture, 4 females/2 males, 68±5 yrs). Samples containing more than 500 erythrocytes per microliter were excluded. CSF was centrifuged at 2,000×g for 10 min to eliminate insoluble material and then stored at −80°C for later biochemical analysis. Brain samples, small pieces of human prefrontal cortex (∼0.2 g), corresponding to cases (3 females/3 males, 63±5 yrs) with no cognitive impairment, no symptoms of any neurodegenerative disease or inflammatory brain disease and not in any acute phases of stroke, were obtained from Banc de Teixits Neurològics, Universitat de Barcelona-Hospital Clínic (Barcelona, Spain). Fragments (∼0.2 g) of normal liver adjacent to colon cancer metastasis (2 females/4 males, 65±7 yrs) were also obtained from the Hospital Clínic of Barcelona. All tissue samples were stored at −80°C until use.

### Tissue Homogenization and AChE Extraction

For AChE extraction, small pieces of frontal cortex or liver stored at −80°C were thawed slowly at 4°C and homogenized (10% w/v) in ice-cold Tris-saline buffer (50 mM Tris-HCl, 1 M NaCl, and 50 mM MgCl_2_, pH 7.4) containing 1% (w/v) Triton X-100 and supplemented with a cocktail of proteinase inhibitors. The suspension was then centrifuged at 100,000×g for 1 hr at 4°C to recover a cholinesterase rich fraction.

Membrane-bound AChE from RBCs was extracted from fresh membrane pellets with 1 M NaCl, 50 mM MgCl_2_, 1% (w/v) Triton X-100 in 15 mM HEPES, pH 7.0 (same volume as the original blood sample, ∼5 mL), supplemented with a cocktail of proteinase inhibitors. After a 2 hr incubation at 4°C, the AChE rich fraction was collected by centrifugation at 100,000×g for 1 hr at 4°C.

### Enzyme Assays and Protein Determination

AChE and BuChE activity were determined by a modified microassay method of Ellman (1961). AChE was assayed with 1 mM acetylthiocholine and 50 µM tetraisopropyl pyrophosphoramide (Iso OMPA), a specific inhibitor of BuChE; while BuChE was measured with 1mM butyrylthiocholine and 10µM BW284c51, a specific inhibitor of AChE. One milliunit (mU) of AChE or BuChE activity was defined as the number of nmoles of acetylthiocholine or butyrylthiocholine hydrolysed per min at 22°C. Protein concentrations were determined using the bicinchoninic acid method, with bovine serum albumin as standard (Pierce, Rockford, IL).

### BuChE Immunodepletion

In plasma and liver samples, BuChE was first immunoprecipitated using an anti-BuChE polyclonal antibody (a generous gift from Prof Oksana Lockridge, University of Nebraska Medical Center, Omaha, NE, USA). Protein A-Sepharose (200 µL of resin) was blocked for 2 hr with 2% bovine serum albumin in PBS and then incubated overnight at 4°C in PBS with 10 µL of rabbit serum containing anti-BuChE antibody. The anti-BuChE affinity resin was incubated with plasma or liver samples (400 µL diluted 1∶3 in PBS) for 8 hr at 4°C, then centrifuged at 500×g and the supernatant fraction was re-incubated with fresh anti-BuChE affinity resin overnight at 4°C. Immunocomplexes were separated by centrifugation and AChE activity was determined in the unbound fraction. These two successive incubations with the anti-BuChE resin ensured that most of the BuChE activity in the samples was removed. Bound and unbound fractions were also examined by Western blotting with the anti-AChE antibody N-19 (Santa Cruz Biotechnology, Santa Cruz, CA).

### Immunoprecipitation of AChE and Binding to Affinity Matrix

Plasma samples immunodepleted of BuChE were incubated with the anti-human AChE antibody MA3-042 (clone HR2; ABR-Affinity BioReagents, Golden, CO) to immunoprecipitate AChE. The resultant plasma AChE was then incubated with an affinity matrix consisting of immobilized fasciculin-2 (Fas2-Sepharose; Fas2 from Latoxan, Valance, France), a polypeptide toxin from snake venom that binds with high affinity to the peripheral anionic sites of AChE [36]. Fas2-Sepharose was prepared as described previously [30], and the binding of plasma AChE to the affinity matrix was tested by incubation overnight at 4°C. Bound proteins were washed and reserved with the unbound fraction for Western blot analysis.

### Sedimentation Analysis

Molecular forms of AChE were separated according to their sedimentation coefficients by centrifugation on 5–20% (w/v) sucrose gradients containing 0.5% (w/v) Triton X-100. Ultracentifugation was performed at 150,000×g in a SW 41Ti Beckman rotor for 18 hr, at 4°C. Approximately 40 fractions were collected from the bottom of each tube and assayed for cholinesterase activities. We defined the ratio of AChE forms G_4_/(G_1_+G_2_) as the proportion of G_4_ molecules versus the sum of the light forms, G_1_ and G_2_. The sucrose fractions containing G_4_ and G_1_+G_2_ peaks were separately pooled, dialyzed against Tris buffer, and concentrated by ultrafiltration (Amicon Ultra 10,000 MWCO, Millipore Corporation, Bedford, MA). AChE species were then assayed by Western blotting and lectin-binding analysis.

### Detection of AChE by Western Blotting

AChE subunits in the different samples were detected by immunoblotting. As plasma samples contain a high amount of certain plasma proteins (albumin, immunoglobulins, transferrin etc.), these proteins were depleted using immunoaffinity-based chromatography (Seppro® IgY14 spin column kit, GenWay Biotech Inc, San Diego, CA) prior to electrophoresis. Samples of plasma (25 µg of protein after protein depletion), liver (50 µg), RBCs (50 µg), frontal cortex (40 µg) and CSF (30 µl) were resolved by electrophoresis on 10% SDS-polyacrylamide slab gels. Following electrophoresis, proteins were blotted onto nitrocellulose membranes, blocked with 5% bovine serum albumin and incubated overnight with different anti-AChE antibodies - N-19 (Santa Cruz Biotechnology), Ab31276 (Abcam, Cambridge, UK), and an antibody raised to the unique C-terminus of human AChE-R [37] (a generous gift from Prof Hermona Soreq, The Institute of Life Sciences, The Hebrew university of Jerusalem, Jerusalem, Israel). The strips were incubated with HRP-conjugated secondary antibodies (Santa Cruz Biotechnology) and immunoreactive AChE was detected using the ECL-Plus kit (Amersham Life Science, Arlington Heights, IL) in a Luminescent Image Analyzer LAS-1000 Plus (FUJIFILM). Molecular weight markers were used to determine protein size (Sigma-Aldrich Co, St Louis, MO). For semi-quantitative analysis, the intensity of AChE bands was measured with the Science Lab Image Gauge v4.0 software provided by FUJIFILM.

### Lectin-Binding Analysis of AChE

Aliquots of plasma and extracts from liver (after BuChE immunoprecipitation), RBCs, total extract from brain frontal cortex and CSF or its enriched G_4_ and G_1_+G_2_ peaks were mixed with immobilized lectins [*Canavalia ensiformis* (Con A) or *Lens culinaris agglutinin* (LCA), both from Sigma-Aldrich Co]. After an overnight incubation at 4°C, AChE-lectin complexes were separated from free AChE by centrifugation at 1000×g for 15 min at 4°C. The unbound AChE activity in the supernatant fraction was used to compare differences in lectin binding among groups.

### Statistical Analysis

Measurements are expressed as means ± SEM. Data was analyzed by Student's t-test or by the Bonferroni test when group means were being compared, using SigmaStat (Version 2.03; SPSS Inc.) software. Statistical significance was designated as *p*<0.05.

## Results

**Figure 1:**
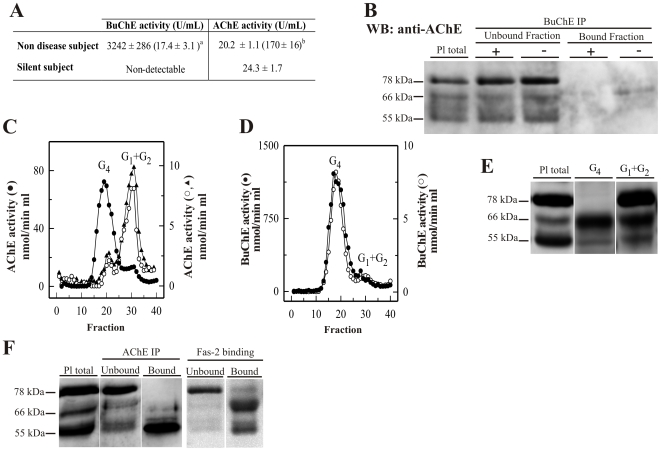
Plasma AChE levels in healthy controls (wild-type) after BuChE immunodepletion and in BuChE-silent individuals. (**A**) Control plasma was immunoprecipitated with anti-BuChE antibody and cholinesterase activity levels determined before^b^ and after^a^ immunoprecipitation (n = 6; 46±4 yrs). AChE activity level in plasma from BuChE-silent individuals is also shown (n = 3; 30±5 yrs). The anti-BuChE antibody does not immunoprecipitate AChE in BuChE-silent plasma (not shown). Values are means ± SEM. (**B**) Immunoprecipitation of control plasma with antibody, followed by immunoblotting with the anti-AChE antibody, N-19. The presence (+) or absence (−) of the anti-BuChE antibody linked to the resin is indicated in the top margin. Prior to electrophoretic analysis, proteins abundant in plasma were depleted by immunoaffinity-based protein subtraction chromatography with IgY microbeads (Seppro™). The anti-BuChE antibody does not immunoprecipitate AChE. Extracts incubated with protein A-Sepharose, in the absence of the antibody, were analyzed in parallel as negative controls. (**C**) Representative profiles of AChE and (**D**) BuChE molecular forms (G_4_ = tetramers; G_1_+G_2_ = monomers and dimers) in control plasma samples before (•) and after (○) BuChE-immunoprecipitation, and in BuChE-silent plasma (▴). (**E**) Representative immunoblot of individual AChE G_4_ and G_1_+G_2_ peak-fractions separated by sucrose gradient centrifugation from control plasma and detected with the N-19 antibody (a similar volume for both the G_4_ and G_1_+G_2_ peaks was loaded in each lane). (**F**) Comparison of the AChE-banding pattern detected with the N-19 antibody, for fractions bound and unbound to the anti-AChE antibody HR2 and to the Fas2-Sepharose affinity matrix.

As the elevated levels of BuChE in human plasma were expected to interfere in the determination of AChE, two cycles of BuChE immunoprecipitation were first performed in plasma aliquots from healthy individuals (46±4 years). Such immunoprecipitation reduced the levels of BuChE ∼190 times, from 3242±286 to 17±3 mU/mL (Fig 1A). We have utilized a polyclonal antibody raised against highly purified human plasma BuChE that has previously been demonstrated to be effective in immunoprecipitating human BuChE [38]. Western blot analysis with the anti-AChE antibody N-19 confirmed the specificity of the immunoprecipitation, with no AChE immunoreactivity detected in BuChE immunoprecipitates (Fig 1B). The remaining AChE activity in the BuChE depleted supernatant was 20±1 mU/mL, much lower than the levels measured before BuChE depletion (Fig 1A).

Both cholinesterases, AChE and BuChE, are expressed as several molecular forms that can be distinguished by their molecular weights and hydrodynamic properties [7]. Plasma supernatants before and after BuChE immunoprecipitation were fractionated on sucrose density gradients to separate the different AChE molecular forms. The G_1_+G_2_ species represented the major peak of AChE activity in the plasma supernatants with only a minor contribution of the G_4_ form after BuChE depletion (Fig 1C). In unprocessed plasma, a major peak observed at the peak of G_4_ AChE, is predominantly the undepleted BuChE activity. This tetrameric peak was significantly reduced after BuChE immunodepletion confirming our observation (Fig 1C). BuChE immunoprecipitation was similarly effective in depleting both major BuChE tetramers and light species (Fig 1D).

We further analyzed the complex AChE banding pattern in an attempt to assign immunopositive AChE bands to specific AChE species and to assess whether the remaining G_4_ peak corresponded only to AChE. Prior to electrophoretic analysis, plasma proteins were depleted by immunoaffinity-based protein subtraction chromatography, as described below. Peaks G_4_ and G_1_+G_2_ from the plasma supernatants fractionated on sucrose density gradients were analyzed by immunoblotting using the anti-AChE antibody N-19 (Fig 1E). This polyclonal antibody was raised against a peptide mapping to the amino terminus of AChE, common to all AChE forms and thus presumably detects all species, including inactive subunits [30]. N-19 detected three major bands of approximately 78, 66 and 55 kDa (Fig 1E). Immunoblotting of the concentrated G_4_ gradient peak demonstrates the presence of tetrameric AChE in the plasma with major 66 and 55kDa bands and a faint 78 kDa band. In comparison, blots of material from the G_1_+G_2_ peaks showed all three AChE bands, similar to that observed in total plasma samples (Fig 1E).

The G_1_+G_2_ forms, which are abundant in plasma, display low binding affinity for several anti-AChE antibodies [14], [29], [30], and inactive AChE molecules have been described as lighter AChE molecules [39]–[41]. We have performed AChE immunoprecipitation with the HR2 antibody and analyzed its pattern of binding. The percentage of AChE not bound to HR2 was 15±2% of total plasma AChE activity. However, when immunoblotted with the N-19 antibody, only the 66 and 55 kDa bands were identified in the bound fraction, while the unbound fraction displayed the three typical AChE bands (Fig 1 F). Similarly, when Fas2 affinity matrix bound protein fraction was analyzed, 66 and 55 kDa bands were identified, with only a faint 78 kDa band; while unbound fractions displayed most of the 78 kDa immunoreactivity (Fig 1 F). The results are in accordance with previous observations in human CSF [30], where a considerable amount of the AChE immunoreactivity (in particular the 78 KDa band) detected by the N-19 antibody was associated with protein that bound poorly to HR2 and to the allosteric AChE inhibitor Fas2. The identity of the 78 kDa band as an AChE subunit was assessed by re-testing our samples with another anti-AChE antibody, ab31276 (see below).

BuChE activity is completely absent in individuals with genetic mutations resulting in a silent phenotype of BuChE (silent BuChE) [42]. These otherwise healthy individuals are a good archetype to study serum AChE activity without interference from BuChE. AChE levels in these individuals varied between 26.7, 25.0 and 21.1 mU/mL, similar to levels found in plasma from normal healthy individuals after BuChE immunoprecipitation (Fig 1A). Sedimentation analysis confirmed that most of the activity corresponded to the G_2_ and G_1_ forms, while tetramers were the minor species (Fig 1C).

**Figure 2:**
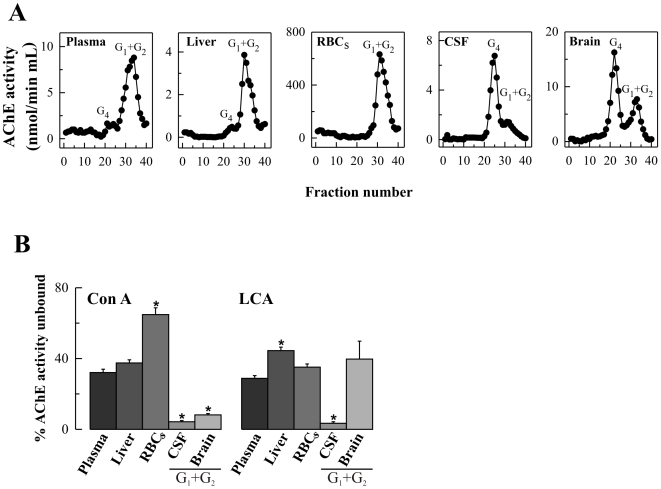
AChE molecular form and lectin-binding profile in human plasma, liver, RBCs, CSF and brain (frontal cortex). (**A**) Representative profiles of AChE molecular forms (G_4_ = tetramers; G_1_+G_2_ = monomers and dimers). (**B**) Comparison of Con A and LCA binding of AChE. Plasma, CSF and total extracts from liver, RBCs and brain (n = 6 for each group) were incubated with immobilized lectins, AChE activity was assayed in the supernatants and the percentage of (%) AChE activity unbound to lectins was calculated. For total CSF and brain extracts, both rich in tetramers, the % AChE unbound to Con A (%Unb Con A_CSF_ = 3±1; %Unb Con A_Brain_ = 4±1) and to LCA (%Unb LCA_CSF_ = 4±1; %Unb LCA_Brain_ = 17±2) were determined. Additionally, individual G_4_ and G_1_+G_2_ fractions, separated by sucrose gradient centrifugation, from CSF and brain extracts (n = 5), were also pooled, dialyzed against Tris-saline-Triton X-100 buffer, and concentrated by ultrafiltration. AChE peaks were then assayed by incubation with immobilized lectins, and the percentages of unbound enzymatic activity were calculated. For CSF tetramers, the %Unb Con A was 0.9±0.5; and the %Unb LCA was 0.3±0.1. For brain tetramers, the %Unb Con A was 1.3±0.2; and the %Unb LCA 2.4±0.2. Please note differences in lectin binding for total AChE from brain or CSF, or its enriched G_4_ fractions, when compared to the respective G_1_+G_2_ peaks (see Figure), revealing distinct glycosylation patterns for different AChE molecules. Values are means± SEM *p<0.05, significantly different from plasma samples, as assessed by one-way analysis of variance with Bonferroni posttest.

The cellular origin of circulating AChE remains controversial. We performed a comparative analysis of AChE from different human tissues and fluids obtained from non-diseased subjects. The different AChE molecular species are cell type-dependent, with differences in developmental and adult tissues and in different species [7]. As the molecular pattern of AChE in human tissues is unclear, we have analyzed the different molecular forms of AChE in liver, RBCs, CSF and brain extracts and compared these to the pattern obtained from plasma which is rich in light species and contains only trace amounts of tetramers (Fig 2A). Sedimentation analysis of AChE from liver (total AChE activity levels, 2.2±0.3 mU/mg) and RBC extracts (491±40 mU/mg) showed similar profiles to that of plasma, whereas CSF (16±2 mU/mL) and frontal cortical extracts (10±2 mU/mg) displayed abundant amounts of G_4_ and small amounts of the G_1_+G_2_ species.

Different cell types have been demonstrated to add different carbohydrate moieties onto AChE [43]. In order to gain further insight into the origin of plasma AChE, we have studied the ability of the plasma enzyme to bind to the lectins Canavalia ensiformis (Con A) and Lens culinaris agglutinin (LCA) in comparison to AChE from liver, RBCs, CSF and brain. Con A binds specifically to mannose groups, while LCA interacts with α-mannosyl residues of N-linked sugar chains, but also requires the presence of a fucose residue bound to the C-6 hydroxyl group of the GlcNAc at the reducing end of glycoprotein, for strong binding. Since different molecular forms of AChE have different glycosylation patterns [9], [44], lectin-binding analysis was performed on total extracts from enriched CSF and the G_1_+G_2_ peaks from brain obtained after sucrose density gradients, focussing on the light forms to compare with the plasma AChE forms (Fig 2A). Similar results were obtained between plasma and liver extracts for the percentage of AChE unbound to Con A. In contrast, a difference in Con A binding was observed between plasma AChE and the G_1_+G_2_ peaks from brain or CSF. The major difference was seen with RBCs where the enzyme from these cells was only poorly recognized by the lectin (Fig 2B). However, a similar interaction with LCA was seen for the AChE from plasma, brain G_1_+G_2_, and RBCs (Fig 2B). The enzyme in plasma does not reproduce the binding pattern of a single tissue, probably due to the diverse number of cellular origins.

**Figure 3:**
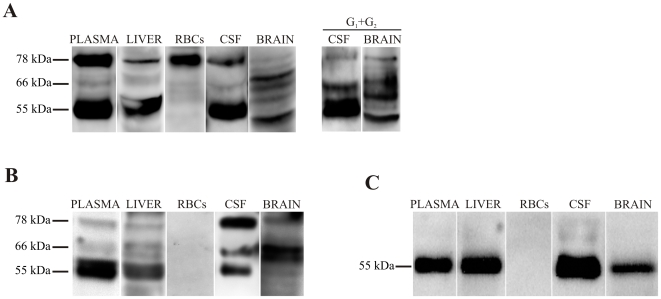
Immunodetection of AChE subunit variants in human plasma, liver, RBCs, CSF and brain (frontal cortex). Samples were immunoblotted with three anti-AChE antibodies, (**A**) the N-terminal N-19, which recognizes all variants; (**B**) the C-terminal ab31276, which recognizes only AChE-T subunits; and (**C**) the anti- AChE-R antibody directed at the unique C-terminus of AChE-R. Representative immunoblot with the N-19 antibody of individual G_1_+G_2_ peak fractions separated by sucrose gradient centrifugation from CSF and brain are included (A, right panel).

Finally, some of the molecular heterogeneity of AChE is also derived from alternative RNA splicing, generating different polypeptide encoding transcripts (called “tailed” or T, “hydrophobic” or H and “readthrough” or R-transcripts) with the same catalytic domain, and distinct C-terminal peptides that determine the ability of the molecule to form oligomers [7], [45]. All transcripts are able to generate AChE monomers, therefore the resultant AChE monomeric variants cannot be distinguished by molecular weight. Thus, we further characterized the complex subunit banding-pattern obtained by SDS-PAGE/Western blotting using different anti-AChE antibodies (Fig 3). As stated above, the antibody N-19 (Fig 3A) raised against a peptide that maps the N-terminus of human AChE, common to all variants, detected the three major bands of approximately 78, 66 and 55 kDa in immunoblots from plasma samples, similar to those from CSF, while brain extracts display a more complex banding pattern with more than 3 bands observed. Immunoblots performed for the G_1_+G_2_ peaks from sucrose density gradients reproduce all the major AChE bands, as observed in total extracts. In liver extracts the 55 kDa band is the only clearly defined band with faint staining for the other heavier subunits. In contrast, blots from RBC extracts showed only a 78 kDa band. In addition to N-19, we used another anti-AChE antibody, ab31276 (Fig 3B), which recognizes residues 601–614 at the C-terminal of human AChE-T. A similar pattern of AChE labelling with N-19 and ab31276 antibodies was also demonstrated for plasma, CSF, brain and liver extracts, indicating that most of the AChE immunoreactivity consists of T-subunits. The rabbit anti-human antibody to AChE-R (Fig 3C) is directed to the unique C-terminus of AChE-R [37]. This antibody detected a 55 kDa band in blots of plasma, liver, CSF and brain indicating the presence of R-subunits; however, T-subunits of this size are also probably present. No band was resolved for the RBC extract with antibodies to AChE-T or R, in accordance with its glycophosphatidylinositol membrane anchor at the C-terminus (Fig 3B, C).

**Figure 4:**
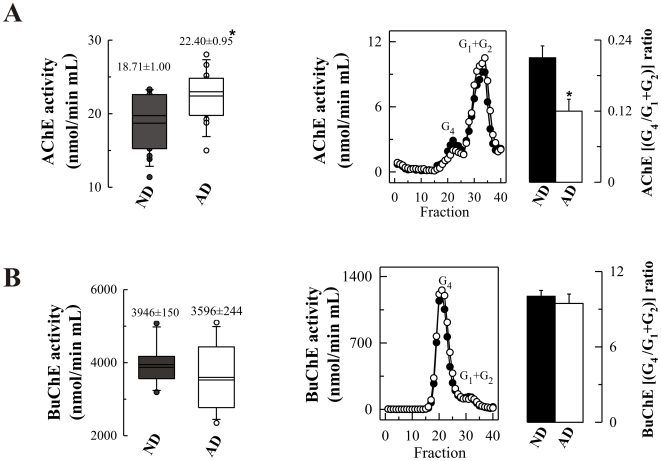
AChE levels and molecular form pattern are altered in plasma samples from non-demented controls (ND) and Alzheimer's patients (AD). (**A**) Box plot comparing total plasma-AChE activity from 15 ND and 14 AD cases, AChE activity was calculated after BuChE depletion. AChE molecular forms were separated, identified by sedimentation analysis (representative profiles; left panel), and a G_4_/(G_1_+G_2_) ratio calculated (n = 6 for each group). * *p*<0.05 significantly different from NDs, as assessed by Student's t-test. (**B**) Total BuChE and molecular form pattern was also calculated, prior to immunoprecipitation, with no statistically significant differences between groups.

The level of plasma AChE in BuChE immunodepleted samples obtained from AD patients were screened for activity. The AChE assay revealed that AD-plasma samples had higher activity, (20% increase; *p* = 0.01), in comparison to age and gender-matched controls, while no change was found in BuChE levels (Fig 4A). To determine if the AChE molecular pattern is altered in AD plasma, the supernatants of BuChE immunoprecipitated samples were fractionated on sucrose density gradients. In AD plasma, a slight decrease was observed in the minor G_4_ peak, and an increase in the G_1_+G_2_ species, resulting in a significant decrease in the G_4_/(G_1_+G_2_) ratio (*p* = 0.006) (Fig 4B). The BuChE levels and G_4_/(G_2_+G_1_) ratio of the AD group were indistinguishable from the control group (Fig 4B).

**Figure 5:**
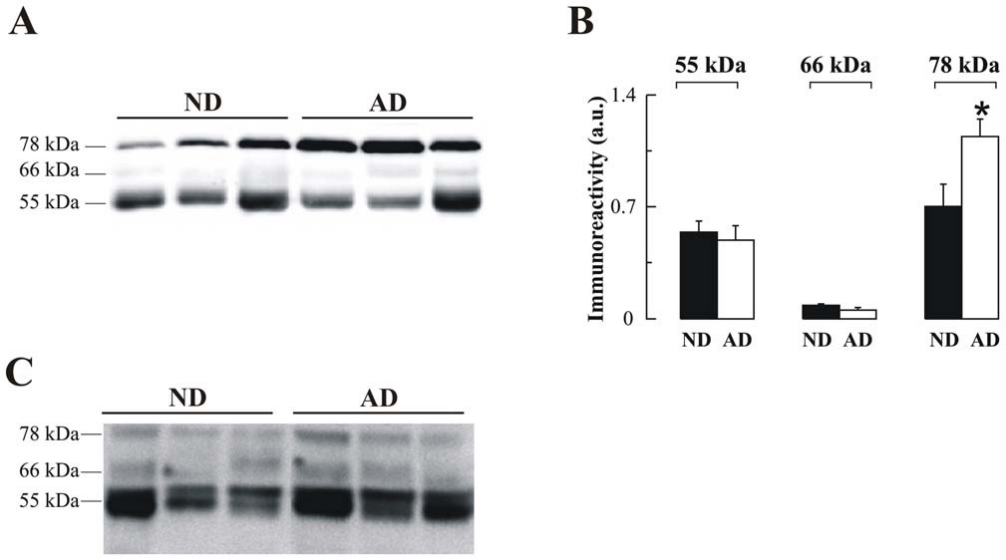
Altered AChE immunoreactivity in plasma of non-demented controls (ND) and Alzheimer's disease (AD) patients. (**A**) Representative blot of plasma-AChE from AD and ND using the antibody N-19, and (**B**) densitometric quantification of the AChE-immunoreactive bands, expressed in arbitrary units (a u.), from 15 ND (▪; n = 15) and 14 AD (□; n = 14) subjects. Proteins abundant in plasma were depleted by immunoaffinity-based protein subtraction chromatography with IgY microbeads (Seppro™) and equivalent amounts of protein were loaded in each lane. Columns represent means ± SEM *p = 0.02 significantly different from NDs as assessed by Student's t test. (**C**) Representative blot of plasma AChE detected with the anti-AChE antibody to the C-terminal, ab31276.

The AD plasma samples were also subject to SDS-PAGE under fully reducing conditions, followed by Western blotting using the anti-AChE antibody N-19. The abundant plasma proteins were depleted as previously described and equivalent amounts of protein were loaded for each sample. N-19 detected the three major bands of 78, 66 and 55 kDa in plasma samples from both AD and control subjects (Fig 5A). Interestingly, the 78-kDa subunit was significantly increased (62%, *p* = 0.02) in AD samples (Fig 5A–B). The large increase in immunoreactivity in comparison with AChE activity may be attributable in part to the partially inactive or subnormally active character of this subunit (Fig 1E). This trend in the increase in immunoreactivity for the 78 kDa band was also observed in blots using the ab31276 antibody (Fig 5C). The levels of other immunoreactive AChE bands were not significantly different in AD plasma (Fig 5B).

## Discussion

There have been an extensive number of investigations in efforts to identify alteration in blood cholinesterase levels as a consequence of disease processes. The status of red blood cell AChE and plasma BuChE are considered important indicators for the treatment of patients affected in several pathologies and in poisoning by organophosphorus compounds; some studies extend this analysis to include plasma AChE. Routinely, when both AChE and BuChE activities are estimated independently within the same extract, an adaptation of the Ellman method (1961) is employed using specific substrates and inhibitors. When measured by this method, plasma BuChE/AChE ratios differ greatly in mammals. Rat or rabbit plasma is reported to have less BuChE than AChE activity (BuChE/AChE∼ 0.5–0.7), while mouse or guinea pig plasma has more BuChE than AChE activity (BuChE/AChE∼ 5–8) (see [46] for a review). In these particular situations, conventional independent measurement of both cholinesterases using specific inhibitors and substrates is reasonably reliable. However, several immuno-chemical studies have indicated that BuChE levels in human plasma are in substantial excess to AChE [21]–[24]. Presently, it is extremely difficult to determine human plasma AChE levels by the use of BuChE inhibitors, as BuChE is presented in much large amounts than AChE. Results of classical spectrophotometric studies of AChE in AD serum or of the enzyme in plasma have been contradictory and vary widely in activity from very low values (6–12 mU/mL), similar to values reported from immunoassays [15], [16], [47], to 10–50 times higher (60–300 mU/mL) [17], [18], to values similar to that observed for BuChE (∼2000 mU/mL) [19], [48]. These large differences are probably due to the use of a diverse concentration of BuChE inhibitors, protocols simultaneously using both competitive AChE and BuChE inhibitors, and the presence of detergents in the plasma dilution buffer (BuChE is highly sensitive to inhibition by detergents; see ref [46], [49]). These inconsistencies are applicable to many previous and current reports of AChE activity in different pathological conditions, and elevated plasma levels of AChE (as high as levels of BuChE) are commonly reported. Previous studies (all from the same group [15], [16], [47]) performed with a conventional Ellman's method have reported plasma AChE activity levels in the range observed in our present study. Interestingly, Atack and colleagues also reported an increase in AChE activity in plasma from AD subjects [15], [16]. In these studies, plasma AChE was assayed in the presence of the BuChE inhibitor iso-OMPA at 10^−4^ M (in our assay, 5×10^−5^ M). Activity was also assayed in the presence and absence of the AChE inhibitor BW284c51, and only the activity sensitive to BW284c51 was considered to represent AChE. Using this methodological approach, the amount of AChE was estimated by subtraction of cholinesterase activity sensitive to the double inhibitor from the activity sensitive to the BuChE inhibitor; and our colleagues reported that more than half (56%) of the plasma AChE activity was constituted by G_4_ tetramers [47]. In this study, we demonstrate that after BuChE immunodepletion, tetramers contribute a minor proportion of the total AChE activity (see Figs 1 and 4). Thus, it is possible that the combination of inhibitors and “subtraction” approach used by Atack et al does not completely eliminate BuChE interference in the determination of AChE levels in human plasma. As such, subtle changes in plasma AChE levels cannot be precisely determined using conventional assay methods.

Our ultimate aim in this study was to determine plasma AChE levels in AD patients in comparison to normal individuals. A prevalence of lighter AChE forms occurring in parallel with a decrease in G_4_ has been observed in both brain and CSF samples in AD [9]. We have also found that the level of a salt-extractable amphiphilic monomeric form of AChE is increased in the brains of transgenic mice which produce the human β-amyloid protein [50], and in the brain and CSF of rats which received intra-cerebroventricular injections of β-amyloid peptides [51]. So far, the exact nature of this subset of G_1_ species which is increased in the AD brain remains unclear, although this minor species can be distinguished from other brain AChE forms (including tetramers but also from other monomeric AChE isoforms), by its unusual lectin-binding pattern and lack of binding to anti-AChE antibodies [14], [30]. AChE exhibits high molecular polymorphism contributed by alternative splicing and by the presence of different carbohydrate moieties on the molecule. Hence, homologous AChE isoforms from different tissues [43] differ in their glycosylation pattern. This observation has been confirmed in the present study. Therefore, it is not surprising that antibodies raised against native AChE species display different affinity for distinct AChE molecules.

In this study, we have been able to determine AChE levels in plasma by immunoprecipitating and therefore removing BuChE. As a result, BuChE levels in the plasma are reduced by ∼190 fold, to lower levels than AChE. Our measured human plasma AChE levels are ∼20 mU/mL, in the range of the activity levels determined for CSF AChE. These levels are also similar to the levels of AChE obtained in the plasma of “BuChE silent” individuals, which serve as useful controls for the specificity of the immunoprecipitation process and for assessing decline in activity due to the precipitation conditions and time of incubation. Furthermore, the possibility of hybrid cholinesterase forms consisting of hetero-multimers of AChE and BuChE subunits has been proposed in normal [52] and pathological conditions [53]. However such possibility has been excluded by western blot analysis for AChE of the plasma BuChE immunoprecipitates, confirming the negligible amount of AChE in such samples.

Using the present methodological approach, based on BuChE immunoprecipitation, we have demonstrated that plasma AChE activity levels are increased in AD patients. This increase correlates with an increase in the light AChE species which are the major species in human plasma, whereas tetramers, which are normally only present in trace amounts, are slightly decreased in AD plasma. This AChE G_1_+G_2_ peak is not a result of any BuChE activity as only minor amounts of light BuChE species remain after immunoprecipitation. Blots of material from the G_1_+G_2_ peak also showed an analogous Western blotting-banding pattern in total plasma. Monomeric AChE was seen in wild-type as well as in silent BuChE plasma by non-denaturing gradient gel electrophoresis [20]. As these light species are the major species in human plasma, this low affinity may explain why previous immunoassay studies observed lower plasma AChE levels than reported in the present study. Similarly, other attempts to separate AChE from BuChE in human plasma have been unsuccessful as the affinity resin usually used to separate these enzymes (edrophonium-Sepharose), binds only soluble tetramers [54], whereas soluble light species present in plasma which have amphiphilic properties do not bind [55]. We have estimated that only ∼20% of the human plasma AChE activity bound to an edrophonium-Sepharose affinity matrix (not shown).

It has been generally assumed that in humans, plasma BuChE originates in liver cells; however contributors to the plasma pool for AChE may include other organs. As AChE is an ubiquitous protein present in many, if not all tissues, it has been proposed that plasma AChE might originate from several tissues, including liver, brain, muscle and nerve, nucleated blood cells and RBCs. All of these organs express the light AChE species which is abundant in human plasma. As sedimentation analysis cannot distinguish between monomeric isoforms which are synthesized in distinct cell types, we have studied the AChE glycosylation pattern, which is cell specific. The ability of lectins to recognize specific carbohydrate residues of glycoproteins, makes them excellent tools to detect subtle differences in glycosylation patterns. We have focused our study on the G_1_+G_2_ species, where we have separated and enriched these peaks in CSF and brain to compare it with the liver, RBCs and plasma, where these forms are abundant. As expected, differences in the binding properties of AChE from liver, brain and RBCs to lectins indicate a distinct pattern of glycosylation derived from different cellular origins of the AChE protein. The comparison of AChE-lectin binding profiles for the two mannose specific lectins, LCA and Con A, give no definitive conclusion because the enzyme in plasma does not reproduce the binding pattern of a single tissue, probably due to the diverse number of cellular origins.

Regulation of AChE at the transcriptional level also corresponds to a cell specific pattern. We have analyzed the protein product of the three different AChE transcripts, with distinct C-terminal peptides, to obtain additional information regarding the cellular origin of plasma AChE. We have performed Western blots and analyzed these plasma samples with anti-AChE antibodies raised against peptides mapping to the common N-terminal or specific C-terminal domains of the various AChE variants. Using western blot analysis, both active and inactive subunits of the enzyme are detected [30], [37]–[41], [44]. Our immunoblotting assays have revealed a complex AChE banding pattern with major AChE bands of 78, 66 and 55 kDa. The full-length AChE is predicted to be ∼70 kDa in size. Accordingly, using non-denaturing polyacrylamide gels stained for AChE activity, molecular weights of AChE subunits are in the range of 70–75 kDa. However, analysis by SDS-PAGE and Western blotting by us and others, under fully reducing conditions and with several different anti-AChE antibodies, detected bands ranging from 75 to 50 kDa, for the AChE protein from different sources and animal species, including humans [30], [37], [44], [56]–[60]. The specificity of the 78 kDa AChE bands and of the lower molecular weight AChE bands was confirmed by immunoprecipitation, Fas2 affinity matrix binding and immunodetection of blots with different anti-human AChE antibodies (including the N-terminal N-19 and the ab31276 antibody, which recognizes the C-terminal residues 601–614 of human AChE-T) and is in agreement with our previous study in human CSF [30]. The lower molecular weight bands are not attributable to differences in glycosylation and may originate by post-translation modification and/or result from the reducing conditions used during electrophoresis. The AChE banding pattern appears specific for each tissue and with no simple relationship to specific molecular forms and enzymatic activity [30], [44].

These data demonstrate that most of the AChE immunoreactivity in the G_1_+G_2_ sucrose fractions from plasma consists of T-subunits. Thus, in our plasma samples, we have attributed the H-subunit to the 78 kDa band; and the 55 KDa band to R-subunits. In addition, bands of 78 and 55 kDa are also attributed to T-subunits as they react with the ab31276 antibody. Thus, T- and R-subunits of these sizes are present in all tissues analyzed except RBCs. The increased level of AChE activity in AD plasma was also accompanied by an increase of 78-kDa subunits. We believe that this increase of the 78 kDa subunit is due in part to AChE-T as blots with the ab31276 antibody also demonstrate increased immunoreactivity for this band in AD samples. Both active and inactive subunits of the protein could contribute to the immunoreactivity of this band. As expected, the 78-kDa band is present in the concentrated G_1_+G_2_ peaks, but little or none was observed in the G_4_ peaks. The 78 kDa T-subunit is also present in G_1_+G_2_ peaks from liver, CSF and brain. Currently our studies can only exclude RBCs as a potential source of the increased AChE in AD plasma. In addition to the 3′ alternatively spliced species of AChE that generate proteins with distinct C termini, the 5′ end is also subject to intricate regulation, as recently demonstrated by Soreq and co-workers [61], generating AChE variants that have extended N-termini (N-AChE-T, N-AChE-R, N-AChE-H). Increased N-AChE expression in the AD brain has been associated with disease progression and apoptotic cell death [62]. The antibodies used in this study do not distinguish between “canonical” and N-extended variants, therefore these variants cannot be excluded from contributing to the complex AChE banding pattern detected in plasma.

Plasma AChE is likely to have multiple cellular origins, including cells from the brain. Thus, we can hypothesize that the increase observed in AD plasma may be associated with the particular increase in the light AChE species which characterizes the AD brain [9], [10].

The transport of circulating molecules in the brain is strictly controlled by the blood-brain barrier. Liposolubility or catalyzed transport are the main modes of passage through the blood-brain barrier. This barrier, offered by capillary endothelial cells of the brain, is highly restrictive for the passage of molecules larger than 500 daltons. The blood-CSF barrier, formed by the choroid plexus and the arachnoid membrane, is a more permeable barrier. Intraperitoneal administration of purified human BuChE to rats demonstrated that a hydrophilic tetrameric form of BuChE crosses the blood-CSF barrier, resulting in less than 0.1% of the BuChE concentration in plasma [38]. It is expected that a higher proportion of a light and amphiphilic protein, such as G_1_ AChE, will cross the blood-CSF barrier. A profound change in blood-brain barrier permeability associated with Alzheimer's disease [63], [64], may be facilitating the movement of AChE monomers through CSF/brain to the blood. Thus, we can presuppose that at least a fraction of the total increase in plasma AChE levels derives from the brain. Further studies of the relationship between CSF/brain and plasma AChE species are still needed.

In conclusion, this is the first study that reports levels of AChE in human plasma without interference by BuChE. We have demonstrated increased plasma AChE activity in subjects with early AD. Although the current observed changes are of insufficient magnitude to warrant the use of AChE as a reliable diagnostic marker due to the substantial overlap between AD and control samples, we can speculate that an assay which is capable of discriminating between plasma AChE variants of different origins will be useful as a potential biomarker. At present, patients included in the study remain clinically diagnosed as probable AD cases and no subjects are neuropathologically confirmed as definite AD cases. Clinical diagnosis of AD, when it is confirmed by post-mortem examination, is found to be about 80–90% accurate at best. Thus, we cannot rule out the possibility that a percentage (10–20%) of the individuals in this group were misdiagnosed. In addition, as the mean age of all controls was approximately 75 years, a small percentage (perhaps 5–10%) of individuals in this group may have early preclinical AD. Therefore, the true degree of overlap between controls and AD cases may be less than that shown, because of the inherent uncertainty of clinical diagnosis. The specificity and sensitivity of plasma AChE as a marker of AD can only be accurately determined in a prospective study, when diagnosis can be confirmed by pathology. Similarly, the effect of long-term treatment with cholinesterase inhibitors on plasma AChE levels should be examined.

Plasma is easily accessible in comparison to CSF, and it is therefore important to continue the measurement of these enzymes in demented patients as an option to CSF. If it is possible to monitor changes in plasma AChE as a function of disease duration and progress, this may provide a new insight into the use of this enzyme as a diagnostic marker in the development of AD pathology.
